# Seed dispersal by carnivores in temperate and tropical dry forests

**DOI:** 10.1002/ece3.7201

**Published:** 2021-02-09

**Authors:** Fabián Alejandro Rubalcava‐Castillo, Joaquín Sosa‐Ramírez, José de Jesús Luna‐Ruíz, Arturo Gerardo Valdivia‐Flores, Luis Ignacio Íñiguez‐Dávalos

**Affiliations:** ^1^ Centro de Ciencias Agropecuarias Universidad Autónoma de Aguascalientes Aguascalientes Mexico; ^2^ Departamento de Ecología y Recursos Naturales Centro Universitario de la Costa Sur Universidad de Guadalajara Autlán de Navarro Mexico

**Keywords:** carnivores, scanning electron microscopy, seed dispersion, temperate forest, tropical dry forest

## Abstract

The seed dispersal mechanisms and regeneration of various forest ecosystems can benefit from the actions of carnivores via endozoochory. This study was aimed to evaluate the role of carnivores in endozoochory and diploendozoochory, as well as their effect on seed viability, scarification, and germination in two forest ecosystems: temperate and tropical dry forest. We collected carnivore scat in the Protected Natural Area of Sierra Fría in Aguascalientes, Mexico, for 2 years to determine the abundance and richness of seeds dispersed by each carnivore species, through scat analysis. We assessed seed viability through optical densitometry using X‐rays, analyzed seed scarification by measuring seed coat thickness using a scanning electron microscope, and evaluated seed germination in an experiment as the percentage of seeds germinated per carnivore disperser, plant species, and forest type. In the temperate forest, four plant species (but mainly *Arctostaphylos pungens*) were dispersed by four mammal species. The gray fox dispersed the highest average number of seeds per scat (66.8 seeds). Bobcat dispersed seeds through diploendozoochory, which was inferred from rabbit (*Sylvilagus floridanus*) hair detected in their scats. The tropical dry forest presented higher abundance of seeds and richness of dispersed plant species (four species) than in the temperate forest, and the coati dispersed the highest number of seeds (8,639 seeds). Endozoochory and diploendozoochory did not affect viability in thick‐testa seeds (1,480 µm) in temperate forest and thin‐testa seeds (281 µm) in tropical dry forest. Endozoochory improved the selective germination of seeds. Nine plant species were dispersed by endozoochory, but only one species (*Juniperus* sp.) by diploendozoochory. These results suggest that carnivores can perform an important ecological function by dispersing a great abundance of seeds, scarifying these seeds causing the formation of holes and cracks in the testas without affecting viability, and promoting the selective germination of seeds.

## Graphical abstract

The Protected Natural Area of Sierra Fría comprises temperate forests and dry tropical forests. We found that the gray fox, coyote, ringtail, coati, and bobcat are the dispersers in the temperate forest and dry tropical forest under study, the latter with a greater abundance and richness of dispersed plant species. In both forests, endozoochory and diploendozoochory did not affect the viability or germination of all seed species. These results suggest that dispersing carnivores adapt to the abundance and richness of seeds in forests where they inhabit, for which they develop important ecological functions by dispersing, scarifying, and promoting the selective germination of thick‐testa seeds in temperate forests and thin‐testa seeds in dry tropical forests.

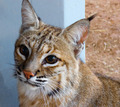

## 1 INTRODUCTION

Endozoochory is a seed dispersal process in which animals consume fruits and subsequently excrete the seeds at varying distances from the parent plant (Cypher & Cypher, 1999; Schaefer & Ruxton, 2011). For seeds dispersed in this manner to survive and germinate, the seed coat must be capable of passing through the digestive tracts of the animals without damage to the embryo (Venier et al., 2012). In particular, carnivorous mammals can be involved in the endozoochory process; they are well known to consume large amounts of fleshy fruits (D'hondt et al., 2011; Harrer & Levi, 2018; Koike et al., 2008) and are capable of dispersing viable seeds (with undamaged embryos) of a wide variety of plant species (Matías et al., 2010). Some carnivores can also disperse seeds via diploendozoochory, which involves the ingestion of seeds by two or more different species of animals in sequence, that is, the seeds pass through a prey and then through a predator or carnivore (Hämäläinen et al., 2017).

Dispersion by endozoochory and diploendozoochory can influence plant distribution patterns (Haarmeyer et al., 2010) by facilitating the establishment of seeds in new habitats (Traveset et al., 2007). The success of both dispersal types and their influence on the recruitment of new plants depend on the number of seeds dispersed by the animals, the survival of these seeds following digestion and their probability of subsequent germination (Schupp et al., 2010; Venier et al., 2012). Therefore, although the absolute number of viable seeds dispersed is an important factor for endozoochory, analysis of the qualitative and quantitative components of dispersion is important to fully understand these processes (Schupp et al., 2010). In this sense, the process of diploendozoochory can be complex because the participation of the carnivore in the second phase of the dispersal process can influence the plant in three ways: transportation of the seeds, alteration of the quantity dispersed, and modification of their viability and germination (Hämäläinen et al., 2017).

Passage of the seeds through the digestive tracts of animals is a critical phase, during which they are subject to several processes that are potentially deleterious to seed viability and germination (D'hondt et al., 2011; Varela & Bucher, 2006). These include wear of the testas and breaking of the physical dormancy period of the hard seeds of some plant species. Passage of the seeds through the digestive tract of animals can modify the seed coat and promote mechanical (in harder coats) or chemical (in softer coats) scarification, increasing the probability of either germination (Peco et al., 2006) or seed death if the embryo has been damaged (Campos et al., 2008). Although the hardness and thickness of a seed coat are important characteristics for seed survival and germination following passage through the digestive tract, the actual effects of this process on the structure of seed coats have been little explored in the literature (Venier et al., 2012). Recently, scanning electron microscopy has been used to observe changes in the seed coat as a consequence of passage through the digestive tract. Schaumann and Heinken (2002) used this technique to observe the testas of *Vaccinium myrtillus*, finding that control seeds presented intact cell walls, while those dispersed by martens (*Martes martes*) presented testas with damaged cell walls. Moreover, Costea et al. (2016) used scanning electron microscopy to observe how seeds of the species *Cuscuta pacifica*, dispersed by birds, presented fragmentation and even complete elimination of their outer layers.

In addition to the viability, change in seed coat and germination analysis in endozoochory, consideration must also be given to the ecosystem in which the seed dispersal takes place. The species of plants and animals that interact through seed dispersal could vary among different forest ecosystems, such as between temperate and tropical dry forests. Temperate forest are important biomes that provide ecosystem services worldwide and their characteristic vegetation type is represented by oaks and pines while, in the region of our study in the Sierra Fría, they are also dominated by *Juniperus* sp. and *Arctostaphylos pungens* (Díaz‐Núñez et al., 2016). The most common seed dispersal modes found in this type of ecosystem are endozoochory (dispersal through consumption by animals), anemochory (dispersal by wind), and epizoochory (dispersal by adhering to mobile living beings) (Willson et al., 1990). Likewise, the seeds found in this type of forest are generally thick testas to protect their embryos from changes in temperature during the seasons and the passage through the digestive tract of dispersing animals (Rubalcava‐Castillo et al., 2020; Ruprecht et al., 2015). Tropical dry forests have canopy cover values greater than 30% and a great variety of flora and fauna (Olson et al., 2000). In particular, the vegetation in the region of the present study is dominated by communities of *Myrtillocactus geometrizans* and *Forestiera phillyreoides* (Argumedo‐Espinoza et al., 2018). The most common dispersal systems found in this ecosystem are endozoochory, anemochory, and hydrochory (dispersal by water) (Correa et al., 2015). The seeds found in this type of forest are generally thin testas to adapt to the shadows under the canopies of the trees (Tiansawat et al., 2014).

The role of carnivorous mammals as dispersal agents has been less studied than that of the birds, primates, and bats in temperate and tropical dry forests (Godínez‐Alvarez et al., 2007; Stoner et al., 2007). The limited studies that focused on the dispersal of seeds by carnivores in forests include that of Rubalcava‐Castillo et al. (2020), who showed the quantitative and qualitative contribution of mammals through endozoochory and diploendozoochory of seeds of *Arctostaphilos pungens* and *Juniperus deppeana* in temperate forest. In turn, Zarco‐Mendoza et al. (2018) described numerous plant species that are dispersed by carnivores in the tropical dry forest. These authors found the seeds of 18 plant species in 384 scats of mammalian carnivores, concluding that carnivores can disperse seeds in abundance and that passage through the digestive tract had positive effects on the germination of two species, neutral effects on six species and negative effects on four species. However, due to the complexity of the diploendozoochoric process, there is a paucity of studies (greater than endozoochory studies) of this mechanism (Rubalcava‐Castillo et al., 2020) in forests worldwide.

This study aims to complement and increase the knowledge of the role played by carnivores in seed dispersal through endozoochory and diploendozoochory by examining all the plant species found in carnivore scats to analyze abundance, viability, seed coat thickness, and germination. Additionally, it aims to evaluate the role of carnivorous mammals in seed dispersal in the two different forest ecosystems: temperate and tropical dry forests. We hypothesize that the carnivorous mammals will perform important ecological functions by dispersing, scarifying, and favoring the germination of seeds with thick testas in temperate forest and seeds with thin testas in tropical dry forest.

## 2 MATERIALS AND METHODS

### 2.1 Study site

**FIGURE 1:**
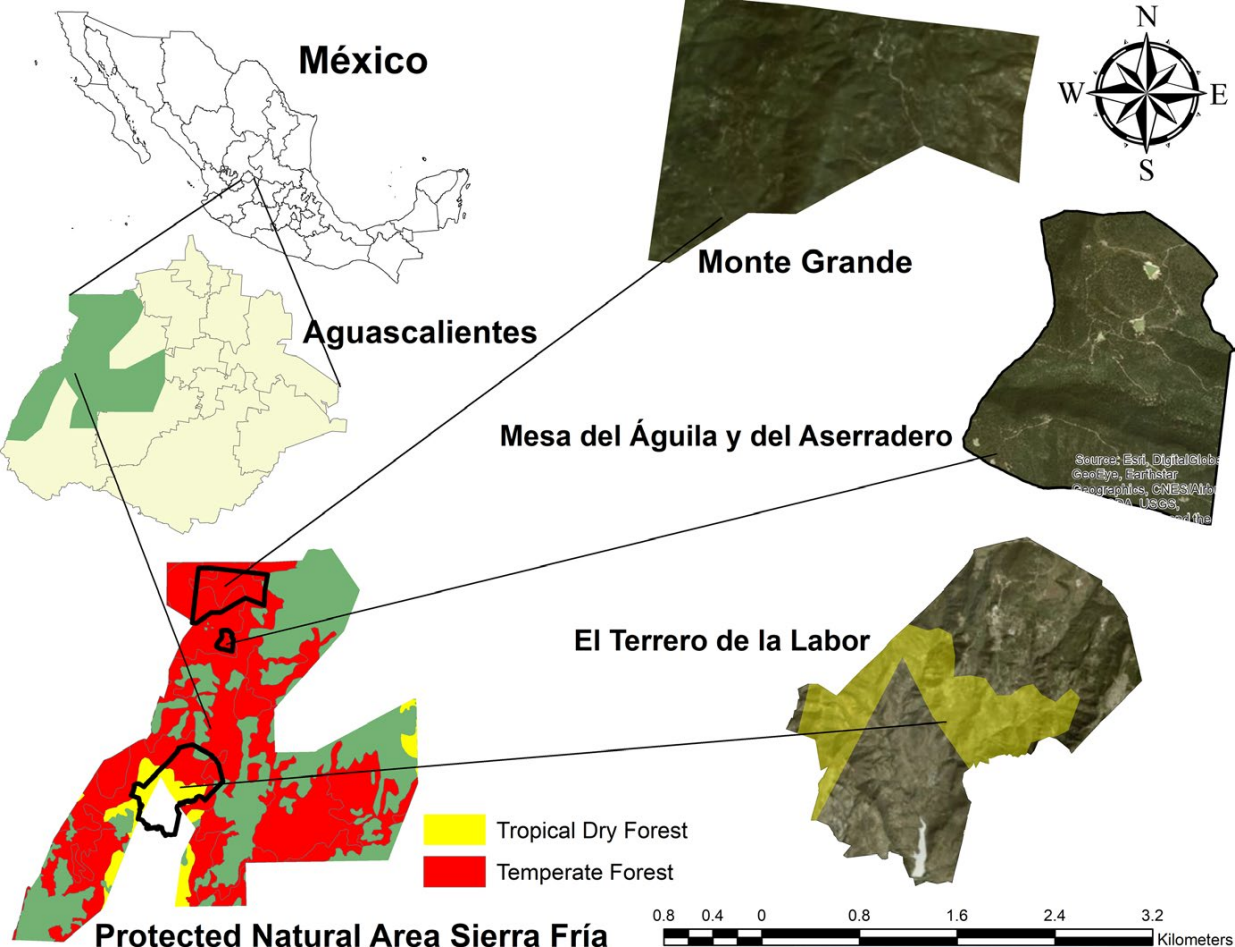
Location of the study areas Monte Grande, Mesa del Águila y del Aserradero, in the temperate forest, and El Terrero de la Labor, in the tropical dry forest of the Sierra Fría Protected Natural Area, Aguascalientes, Mexico

We conducted the study in two temperate forest areas and one tropical dry forest area in the Protected Natural Area of Sierra Fría (PNA‐SF) that host 14 species of carnivorous mammals (Chávez‐Andrade et al., 2015), which is located in the western zone of the state of Aguascalientes, in Mexico (Figure 1). The temperate forest has a temperate subhumid climate and presents summer rainfall (Rzedowski, 1978) with an average annual precipitation of 650 mm (SEDESO, 1995). The natural plant communities in this forest are composed of pointleaf manzanita or pingüica (*Arctostaphylos pungens*), checkerbark juniper or táscate (*Juniperus deppeana*), strawberry tree or madroño (*Arbutus* sp.), oak (*Quercus potosina*), Chihuahua pine or ocote chino (*Pinus leiophylla* var. Chihuahuana), and twisted leaf pine or pino colorado (*Pinus teocote*), among others (Díaz‐Núñez et al., 2016). On the other hand, the tropical dry forest has an average annual precipitation of 625 mm (Sosa‐Ramírez, 1998), and the plant communities are composed of blue myrtle‐cactus (*Myrtillocactus geometrizans*), palo bobo (*Ipomea murucoides*), kidneywood tree (*Eysenhardthya polystachya*), torchwood copal (*Bursera fagaroides*), and palo blanco (*Forestiera phillyreoides*), among others (Argumedo‐Espinoza et al., 2018). We searched for scats in two temperate forests sites: “Monte Grande” (961 ha) and “Mesa del Águila y del Aserradero” (527 ha) and in one tropical dry forest site: “El Terrero de la Labor” (1,227 ha) (Figure 1). We sampled three different transects on each visit for each study area.

### 2.2 Collection and identification of scats

We conducted field visits in each site once a month throughout the years of 2018 and 2019. Within each site, we collected scats located by sight within transects (Nova, 2012) of following walking routes through the study area to locate scats by sight. Each transect is delimited by a central line of 2 km in length, with two parallel lines located at a distance of 20 m on either side of the central line (Rubalcava‐Castillo et al., 2020). We have established a total of 30 transects in each area on trails for the movement of fauna, on dirt roads and among vegetation, that is, the scats were screened over a total area of 2 (temperate, tropical dry forest) × 30 (replicate transects) × 2,000 m (transect length) × 40 m (transect width/buffer). We collected all of the scats found within the transect for identification, except for those that were dry and old, with a gray coloration, to avoid bias in the data related to the collection of old scats and a preference of collection toward specific animals. Thus, each transect was sampled twice during the study period. Scats corresponding to each mammalian species were identified based on the *Manual for Tracking the Wild Mammals of Mexico* (Aranda‐Sánchez, 2012). Each scat associated with the mammalian species was classified into two categories according to the dietary habits of the animal: (a) mesocarnivorous/endozoochorous animals, the diet of which includes meat, insects, fruits, fungi, and other plant elements, for example, the gray fox, and (b) hypercarnivorous/diploendozoochorous animals, the diet of which is based on meat (prey) and carrion, for example, the felines.

### 2.3 Identification and abundance of seeds

We counted and identified all the seeds from the collected scats. We left the scats to dry at room temperature (23°C) for 24 hr in Petri dishes and then extracted the seeds using a sieve (1 mm mesh size) to retain the smallest seeds and wash them with running water. Once separated, the seeds were left to dry for 24 hr before analysis and identification. We used a stereoscopic microscope (Leica Microsystems, MZ6, Switzerland) to quantify the total number of seeds per scat. We also identified the seed species present in each scat to determine the richness using keys for each species (Rzedowski & Rzedowski, 2005) and comparisons with specimens from the Herbarium of the Autonomous University of Aguascalientes. We used these data to determine species richness, that is, the number of seeds per scat per plant species, for each animal disperser and forest type (temperate or tropical). To infer diploendozoochory by the hypercarnivorous mammals, we also identified other elements contained in the scat. In the particular case of bobcats, which are fully carnivorous/predator (Sánchez‐González et al., 2018), if the presence of seeds associated with prey hairs is verified in the same scat, we identified the species of the seed and, in turn, the species of the prey, through the guard hairs. We then conducted a bibliographic review to verify whether the species of the seeds found form part of the diet of the prey. To determine the potential prey of the hypercarnivores, we identified the guard hairs of the prey contained in the scats of the predators by consulting the identification guide developed by Monroy‐vilchis and Rubio Rodríguez (2014).

### 2.4 Three‐step procedure

We used a three‐step procedure to generate a robust evaluation of how ingestion by carnivores affects testa wear and seed viability and germination for each forest type and animal seed disperser and dispersed plant species. Each step consists of a test: (a) a viability test, to determine whether the embryos from dispersed seeds show damage or remain intact, (b) a test of wear in the thickness of the testas using scanning electron microscopy, and (c) a germination test to determine the impacts of the passage of seeds through the digestive tracts of the carnivores on seed germination. To carry out these tests, according to the abundance (seed per scat) per plant species, the seeds of five plant species (three temperate species and two tropical species) were used. Three replicates of 30 seeds (90 seeds) were used for each plant species with its respective disperser. In a few cases, the total of 30 seeds could not be collected, so only the available quantity was evaluated. The same batch of 90 seeds for each treatment was used in the viability and germination tests. For the scanning electron microscopy test, a representative plant species was selected for each forest, according to the abundance of seeds and the number of mammals that dispersed it. Thus, two seeds per mammal were analyzed.

### 2.5 Control groups

To check the effects of endozoochory and diploendozoochory on seeds from carnivores versus seeds from the canopy, we established control groups based on the abundance of seeds found in the scats for each plant species. During the spring of 2019 (March‐June), we collected 100 seeds from the canopy of 12 random individuals (trees) with ripe fruits for each plant species from the study areas to conduct compare viability, testa thickness, and germination tests with the defecated seeds.

### 2.6 Viability test

We carried out viability tests for both the control and defecated seeds through optical densitometry analysis using X‐ray equipment (Faxitron X‐Ray Corporation, Texas, USA, at 10 s and 26 kV intensity). We performed densitometry analysis for each individual seed from the controls and the scats of each mammal, dispersed plant species and forest type, based on the technique proposed by Rubalcava‐Castillo et al. (2020), which consists of observing the radiograph and distinguishing the viable seeds with undamaged testas and embryos from the nonviable seeds, by the presence of underdeveloped/incomplete embryos, empty seeds, or no embryo.

### 2.7 Test of wear in testa thickness

**FIGURE 2:**
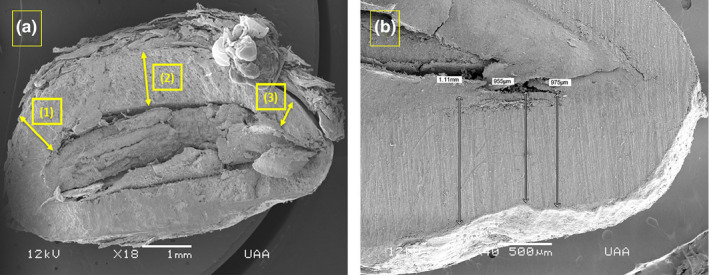
Image (×18) of a seed of *Juniperus* sp. taken with a scanning electron microscope. (a) Seed sections are shown: (1) section opposite the micropyle, (2) central section, and (3) the section on the side of the micropyle, (b) where the three measurements were taken for analysis

We used whole and sagittal cut seeds for this test. We coated the seeds with yellow gold for 4 min in a Denton Vacuum apparatus (JFC‐1100®, JEOL LTD, Tokyo, Japan). Once prepared, we placed the seeds inside the camera of a scanning electron microscope (JSM‐35C®, JEOL LTD, Tokyo, Japan) (Dykstra & Reuss, 2003) in the Electron Microscopy Laboratory at the Autonomous University of Aguascalientes. We observed the seeds inside the camera and took thickness measurements on three parts of the testa at magnification 40×: (3) the micropyle portion, (2) the central portion, and (1) the portion opposite the micropyle (Figure 2a), in which three measurements were taken per portion (Figure 2b). In addition, we recorded the qualitative characteristics of the surface and interior of the testas, including loss of superficial plant layers and the presence of holes and cracks in the external and internal parts of the testas.

### 2.8 Germination test

The control (canopy) seeds were stored for 1 month and subsequently used for the germination test. We washed the seeds from the scats and the canopy seeds in a 10% chlorine solution at a concentration of 50 ml per 500 ml for 30 s in order to avoid absorption of the chlorine into the seed to clean the seeds of bacteria and fungi. Subsequently, we carried out three washes (30 s each wash) with distilled water to eliminate excess chlorine, and finally we applied fungicide to each seed (Interguzan 30–30, Int. Química de Cobre, Mexico) at a concentration of 1.2 g per 100 ml to prevent fungal growth. We placed the seeds in Petri dishes, carrying out three replicates (one petri dish per replicate) of 30 seeds per treatment. The experimental units consisted of 90 mm × 15 mm petri dishes, in which we sowed 30 seeds on filter paper and cotton, which were immediately moistened with 4 ml of distilled water (Antonio‐Bautista, 2012). We placed the seeds inside a germination chamber (Lab Line, Imperial III, Melrose Park, IL, USA) at a controlled temperature of 25°C and a photoperiod of 12 hr for 61 days. All dishes were monitored every 2 days, recording the number of germinations per sample, in which germination was considered to have occurred when the radicles became visible (Herminio, 2003).

### 2.9 Statistical analysis

To analyze the contribution of each mammal to the dispersion of seeds (abundance of seeds) for each plant species in the temperate and tropical dry forest, we generated two different datasets for each forest type. Each dataset consisted of the number of seeds per scat for each carnivore and dispersed plant species, where the variable to be analyzed was the number of seeds per scat. Therefore, the sample size was dependent on the number of scats found for each animal species and plant. A multivariate GLM analysis was performed for each of the seed variables: abundance, species richness, viability, wear thickness, and germination, to determine significance in the combination of factors: (A) seed treatment (defecated versus control), (B) animal species, and (C) type of forest (temperate versus tropical). The three factors were included in the same model as predictors. For temperate and tropical dry forest, we performed a Kruskal–Wallis test to determine significant differences among the average abundances of seeds per scat dispersed by each mammal under the null hypothesis that there will be no differences between the abundances of seeds per scat dispersed among carnivores. For the viability tests, testa thickness by SEM and germination, Dunnett's test was used, where the variables analyzed were the average percentage of viability and germination and the average testa thickness (µm). We conducted the above to determine significant differences between the average percentages/thicknesses in the seeds dispersed by each carnivore compared to the seeds of the canopy (controls) for each plant species selected. We used the null hypothesis that there will be no differences between the average percentages/thicknesses of the seeds dispersed by each carnivore and their respective controls. We conducted all analyses at a significance level of 95%, using the Statgraphics program (16.1, 2012), with the values expressed as the average number of seeds per scat/% of viability or germination/testa thickness (µm): $\bar{x}$ ± *SD*.

## 3 RESULTS

### 3.1 Seed dispersal

According to the GLM analysis, the combined effect of the factors (mammal species and forest type) is significant for the abundance of seeds (*R* ^2^ *= *61.4%, *F*_13,86_ = 10.55, *p* = .0001). Four species of mammals dispersed seeds in the temperate forest: gray fox, coyote, ringtail, and bobcat. These species dispersed four species of plants: *Arbutus* sp., *A. pungens*, *Juniperus* sp., and *Yucca* sp. (Table 1), with significant differences found in the average abundance of seeds (seeds per scat) dispersed in the scats among the four mammalian species (*X* ^2^ = 14.73, *p* = .002). The gray fox dispersed the highest average number of seeds (seeds per scat: $\bar{x}$ ± *SD* = 66.8 ± 68.2), particularly those of the species *A. pungens* (241 ± 106) and, in turn, spread the highest number of scats, with the seeds of *Juniperus* sp. presenting the greatest frequency of appearance since they were found in 50 of the scats. The gray fox was also the most efficient mammal in seed dispersal in this system since 100% of its scats contained seeds. We identified the strictly carnivorous/hypercarnivorous bobcat as a diploendozoochorous species because all the seeds found in their scats were with the hair of the rabbit species *Sylvilagus floridanus* (11.0 ± 0) (Table 1).

**TABLE 1:** Data for each forest type and carnivorous mammal species, describing the total number of scats, percentage of scats with seeds, and average abundance of seeds ($\bar{x}$ ± *SD*) for each plant species

Forest	Disperser	Seed species	Scats (*N*)	Scats with seeds (%)	Abundance of seeds (seeds per scat: $\bar{x}$ ± *SD*)
Temperate	Gray fox	** **	55	100.0	66.8 ± 68.2
		*Arctostaphylos pungens*	5		241.0 ± 106.0
		*Juniperus* sp.	50		51.3 ± 30.9
		*Yucca* sp.	2		18.5 ± 21.9
	Coyote	* *	11	27.3	4.0 ± 2.7
		*Arctostaphylos pungens*	1		2.0 ± 2.8
		*Juniperus* sp.	2		3.0 ± 2.9
		*Yucca* sp.	1		8.0 ± 2.10
	Ringtail	* *	9	77.8	43.4 ± 58.2
		*Arbutus* sp.	2		128.0 ± 10.0
		*Juniperus* sp.	3		13.67 ± 7.02
		*Yucca* sp.	2		3.5 ± 0.7
	Bobcat	* *	9	11.1	11.0 ± 0.0
		*Juniperus* sp.	1		11.0 ± 0.0
Tropical	Gray fox	* *	6	83.3	505 ± 1,002.0
		*Celtis* sp.	1		84.0 ± 0.0
		*Forestiera phillyreoides*	2		44.5 ± 26.2
		*Myrtillocactus geometrizans*	1		2,297.0 ± 0.0
		*Prosopis laevigata*	1		54.0 ± 0.0
	Ringtail	* *	22	81.8	916.0 ± 1,637.0
		*Forestiera phillyreoides*	5		23.2 ± 29.8
		*Myrtillocactus geometrizans*	13		1,588.0 ± 1,945.0
		*Prosopis laevigata*	1		12.0 ± 0.0
		*Solanum* sp.	4		72.3 ± 56.1
	Coati	* *	3	66.7	8,639.0 ± 12,203.0
		*Myrtillocactus geometrizans*	1		17,267.0 ± 0.0
		*Prosopis laevigata*	1		10.0 ± 0.0
	Badger	* *	2	50.0	7.0 ± 0.0
		*Prosopis laevigata*	1		7.0 ± 0.0

Note

The occurrence of each plant species in the number of scats (*N*) is shown.

Four mammals dispersed seeds in the tropical dry forest—the gray fox and the ringtail, as in the temperate forest, as well as the coati and the badger. However, we found no diploendozoochoric mammals (Table 1), since it was not possible to find scats of hypercarnivores, and all the scats containing seeds in this forest were composed entirely of remains of fruits and their respective seeds. These were considered as endozoochoric according to the criterion proposed in the methodology and therefore diploendozoochory was discounted. We found no significant differences in the average abundance of seeds (seeds per scat) dispersed in the scats among the four mammalian species (*X* ^2^ *= *1.88, *p* = .59); however, the coati dispersed the highest average abundance of seeds (seeds per scat: $\bar{x}$ ± *SD* = 8,639 ± 12,203) in only three of their scats found. Thus, the tropical dry forest presented a greater abundance and richness of dispersed species (*R* ^2^ *= *15.41%, *F*_6,93_ = 2.82, *p* = .01), since five plant species were dispersed. The gray fox and the ringtail dispersed four plant species in 83% and 81% of their scats, respectively (Table 1).

### 3.2 Viability of seeds dispersed by carnivores

Five species of plants were selected for this analysis, based on the abundance of seeds found in the scats as specified in the methodology. For the temperate forest, we used seeds of *Arbutus* sp., *A. pungens*, and *Juniperus* sp., while for the tropical dry forest, we used seeds of *F. phillyreoides* and *M. geometrizans*. According to the GLM analysis, the combined effect of the three factors (mammal species, forest type, and seed treatment) was significant for the viability of seeds (*R* ^2^ *= *1.33%, *F*_6,3,658_ = 8.26, *p* < .0001). In seeds of *Arbutus* sp. in the temperate forest, we found no significant differences (*F*_1, 4_ = 2.40, *p* = .19) in the average percentage of viability ($\bar{x}$ ± *SD*) of the seeds dispersed by the ringtail (70.0 ± 19.8%), compared to those of the control (86.0 ± 7.7%). For *A. pungens*, we found significant differences (*F*_2, 7_ = 11.96, *p* < .0001) in the average viability of the seeds dispersed by each mammal with respect to the control, particularly in those dispersed by the gray fox (91.6 ± 6.1%), which presented the highest percentage of viability compared to the control (76.0 ± 5.7%). In *Juniperus* sp., we found no statistical differences (*F*_4, 53_ = 2.20, *p* = .08) in seed viability for each mammal with respect to the control. Once again, however, the seeds dispersed by the gray fox (82.6 ± 12.4%) had a higher percentage of viability than those of the control (77.0 ± 6.0%), while practically half of the seeds dispersed by the bobcat were viable. In the tropical dry forest, for *F. phillyreoides* seeds, we recorded no significant differences in the average percentages of seed viability for each mammal, with respect to the control (*F*_2, 8_ = 0.57, *p* = .58). However, the seeds dispersed by the gray fox (92.0 ± 11.3%) presented the highest percentage of viability compared to the control (79.0 ± 6.8%). Likewise, for *M. geometrizans*, we found no significant differences (*F*_3, 14_ = 1.10, *p* = .38), although the viability of the seeds dispersed by most mammals achieved a higher percentage compared to the control (87.0 ± 3.8%), apart from those dispersed by the ringtail (75.3 ± 14.6%), which presented a lower percentage of viability (Table 2).

**TABLE 2:** Average viability percentages ($\bar{x}$ ± *SD*) from X‐ray optical densitometry of seeds of *Arbutus* sp., *Arctostaphylos pungens*, and *Juniperus* sp., with their respective animal dispersers, in the temperate forest; and of seeds of *Forestiera phillyreoides* and *Myrtillocactus geometrizans*, with their respective animal dispersers, in the tropical dry forest (both forests located at the Sierra Fría PNA in Aguascalientes, Mexico)

Forest	Seed species	Disperser species	Seeds (*N*)	Viability (%)
Temperate	*Arbutus* sp.	Ringtail	90	70.0 ± 19.8
		Control (canopy)	90	86.0 ± 7.7
	*A. pungens*	Gray fox	90	91.6 ± 6.1*
		Coyote	2	67.0 ± 0.0
		Control (canopy)	90	76.0 ± 5.7
	*Juniperus* sp.	Gray fox	90	82.6 ± 12.4
		Coyote	6	67.0 ± 0.0
		Ringtail	40	79.3 ± 15.1
		Bobcat	11	54.5 ± 0.0
		Control (canopy)	90	77.0 ± 6.0
Tropical	*F. phillyreoides*	Gray fox	90	92.0 ± 11.3
		Ringtail	90	84.0 ± 18.1
		Control (canopy)	90	79.0 ± 6.8
	*M. geometrizans*	Gray fox	90	82.0 ± 0.0
		Ringtail	90	75.3 ± 14.6
		Coati	90	90.0 ± 0.0
		Control (canopy)	90	87.0 ± 3.8

Note

*N* indicates the maximum number of seeds per treatment.

* Statistically significant differences according to the Dunnett test (*p* < .05).

**FIGURE 3:**
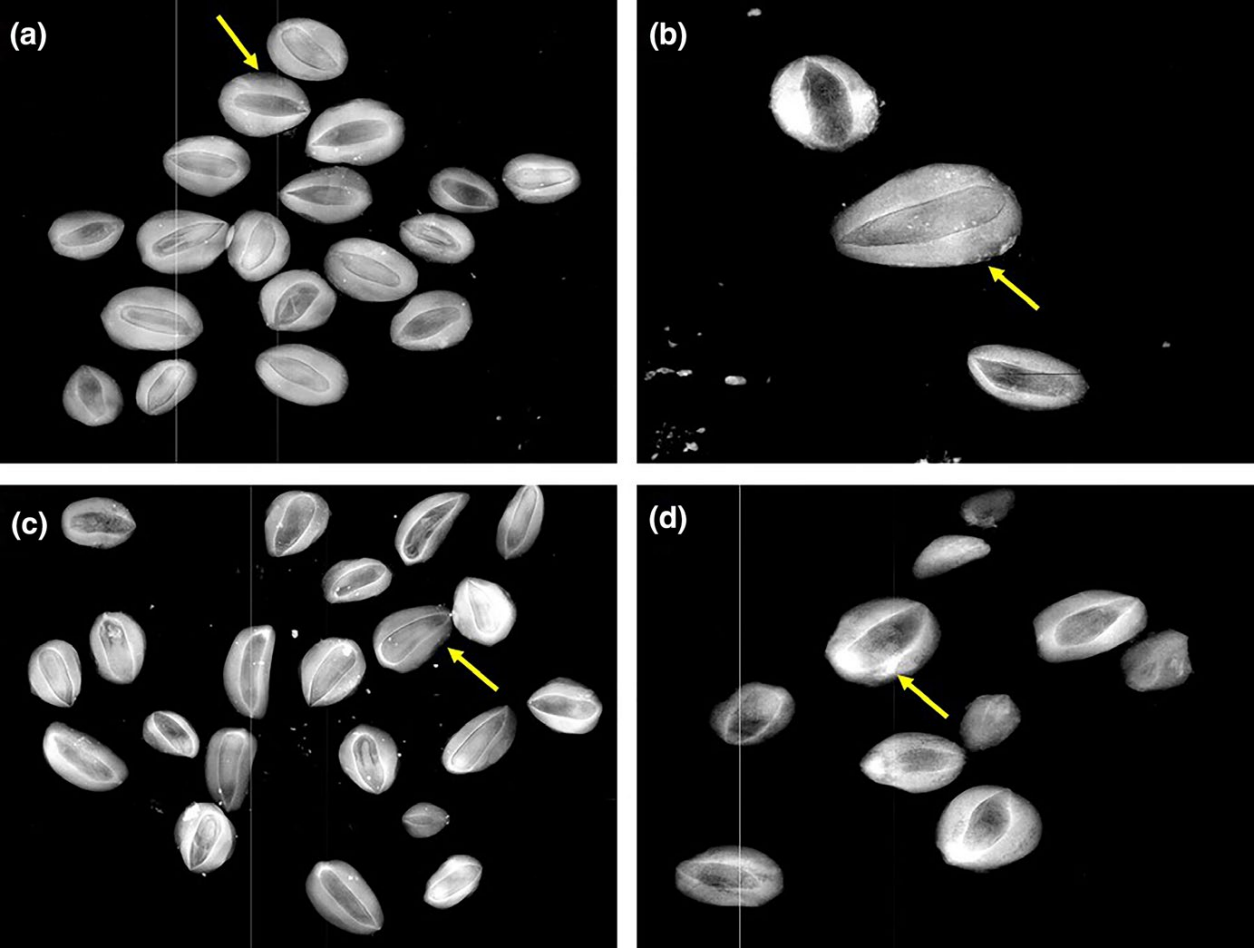
Seeds of *Juniperus* sp. from X‐ray optical densitometry of the seeds from the scats of the different mammals in the temperate forest of the Sierra Fría PNA in Aguascalientes, Mexico. Seeds dispersed by (a) gray fox, (b) coyote, (c) ringtail, and (d) bobcat

As part of the seed viability analysis, physical changes were observed in the testas of the selected seeds, that is, in the seeds with their respective dispersers (Table 1) of *Arbutus* sp., *A. pungens* and *Juniperus* sp. for temperate forest and *F. phillyreoides* and *M. geometrizans* for tropical dry forest. When observing the radiographs for each selected plant species with their respective dispersers, particularly in the seeds of *Juniperus* sp., we observed apparent changes to the seed testas due to mechanical damage during mastication, and due to passage through the digestive tracts of the gray fox (Figure 3a), coyote (Figure 3b), ringtail (Figure 3c), and bobcat (Figure 3d), but with no apparent damage to the seed embryos. Damage to the outer layers of the testa therefore had no influence on viability.

### 3.3 Wear in testa thickness

**FIGURE 4:**
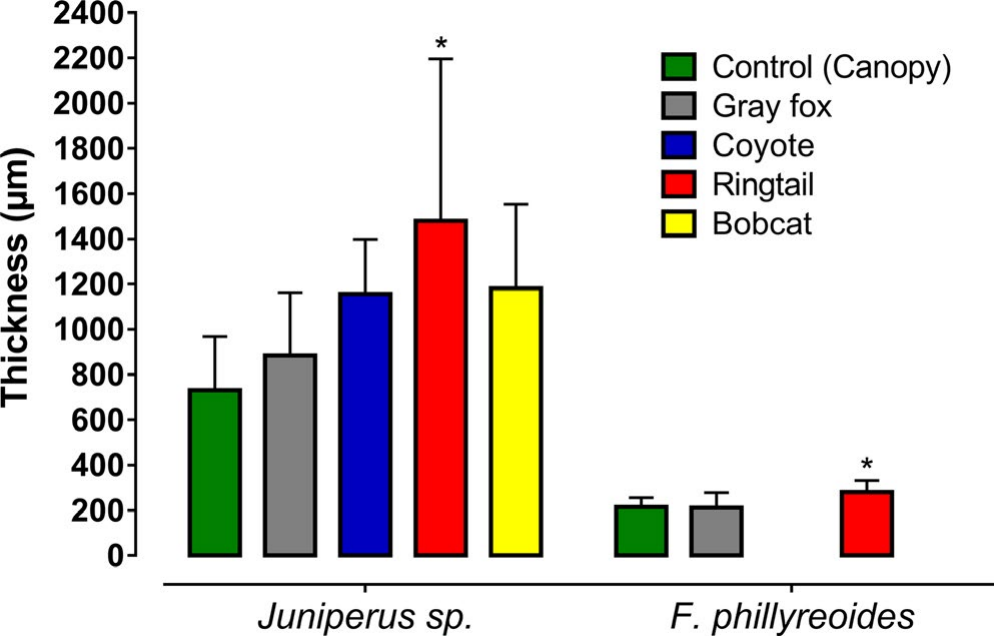
Measurements (µm) of the average thickness (±*SD*) of the seed testas of *Juniperus* sp. in the temperate forest and of the seed testas of *Forestiera phillyreoides* in the tropical dry forest, using seeds obtained in the field from scats of endozoochoric and diploendozoochoric mammals, and from the canopy in the Sierra Fría PNA in Aguascalientes, Mexico. *Statistically significant differences according to the Dunnett test (*p* < .05)

The thickness of the testas of seeds of *Juniperus* sp. from temperate forest and *F. phillyreoides* from tropical dry forest was analyzed. According to the GLM analysis, the combined effect of the three factors (treatment, mammals, and forest) was significant for the wear in the testa thickness of the seeds (*R* ^2^ *= *62.19%, *F*_6,61_ = 16.72, *p* < .0001). For *Juniperus* sp., significant differences were found in the average thickness of the seed testas for each mammal, compared to the control (*F*_4.40_ = 4.47, *p* = .00), since for all the mammals, the digested seeds presented greater thicknesses ($\bar{x}$ ± *SD*) than the control (731 ± 238 µm), particularly in the ringtail (1,480 ± 717 µm), which presented the highest average. In tropical dry forest, the thickness values were lower in the control and for all the dispersers, compared to those of the temperate forest; however, significant differences were obtained between the seeds dispersed by each mammal and those of the control (*F*_2,20_ = 4.30, *p* = .02), since the seeds dispersed by the ringtail had the highest average thickness (281 ± 50.6 µm) relative to the control (215 ± 42.3 µm) (Figure 4).

**FIGURE 5:**
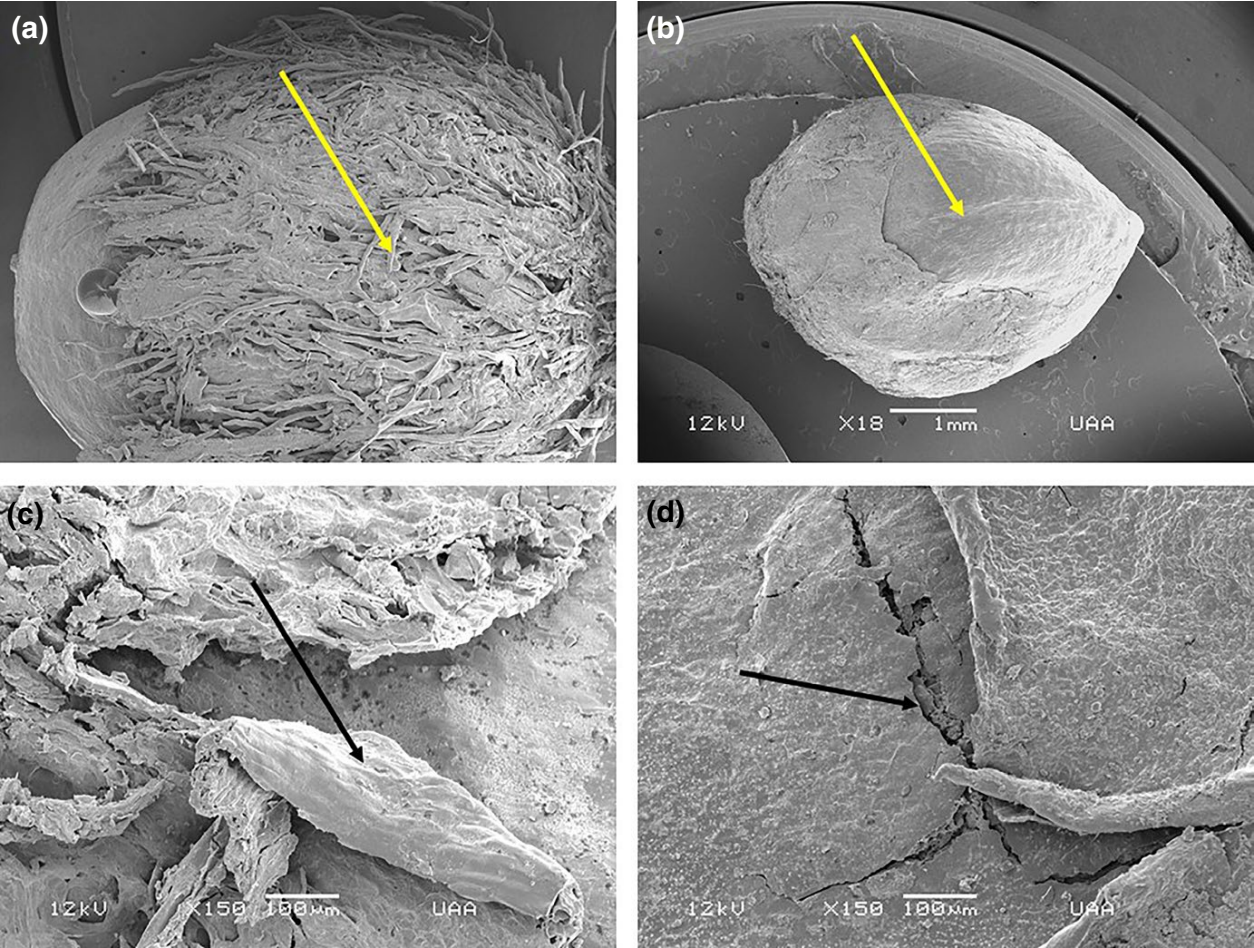
Images of seeds of *Juniperus* sp. of the temperate forest in the Sierra Fría PNA in Aguascalientes, Mexico, obtained using scanning electron microscopy. (a) Control seed with the protective outer fibrous layer intact (×19). (b) Seed dispersed by the bobcat with the outer layer removed (×18). (c) Seed dispersed by the gray fox with the testa surface detached (×150). (d) Seed dispersed by the ringtail with cracks across the testa (×150)

For the control in the temperate forest, the testa of the *Juniperus* sp. seed had a protective external vegetal fibrous layer (Figure 5a), which may have been removed when the seed passed in a first stage through the digestive tract of the rabbit and/or in a second stage through that of the bobcat (Figure 5b). This layer also presented wear or removal when the seed passed through the gray fox gut (Figure 5c) and the formation of cracks could be seen following passage through the tract of the ringtail (Figure 5d). These changes occurred in all of the seeds of each disperser although, due to time and budget, only two seeds per mammal were analyzed by scanning electron microscopy.

**FIGURE 6:**
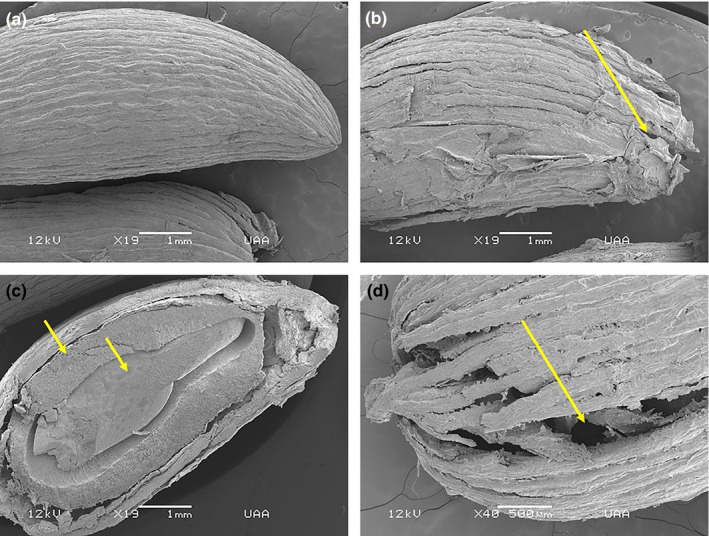
Images of seeds of *Forestiera phillyreoides* from the tropical dry forest in the Sierra Fría PNA in Aguascalientes, Mexico, obtained using scanning electron microscopy. (a) Control seed with a striated layer (×19). (b) Seed dispersed by the gray fox with cracks and holes on the outer layer (×19). (c) Seed dispersed by the gray fox with no damage to the endosperm or the embryo (×19). (d) Seed dispersed by the ringtail with large holes (×40)

In the tropical dry forest, the seeds of *F. phillyreoides* did not have this protective layer, but only a line pattern presented on the surface (Figure 6a). There was a change in the pattern of these lines when they passed through the gray fox, presenting cracks and holes (Figure 6b) but with no damage to the internal parts, and the endosperm and embryo therefore remained in good condition (Figure 6c). We observed large openings on the surfaces of the seeds from the ringtail scats (Figure 6d). As in the temperate forest, these characteristics were presented in all the seeds of each disperser; therefore, in four seeds from two mammals, these changes in the testas were seen.

### 3.4 Germination

The five plant species with their respective dispersers selected for the X‐ray analysis were also used for the germination test. According to the GLM analysis and as happened in the variable seed passes, the combined effect of the three factors was significant for seed germination (*R* ^2^ *= *8.78%, *F*_6,1,218_ = 19.55, *p* < .0001). The seeds obtained from the coyote and bobcat scats did not germinate, producing a germination rate of 0%. We therefore based the analysis on the three remaining mammal species (gray fox, ringtail, and coati) and the controls of the five plant species (Table 3). When performing the analysis for each plant species, we found no significant difference for *Arbutus* sp. in the temperate forest (*F*_1,4_ = 1.58, *p* = .27), since the average germination percentages (±*SD*) of seeds from the control (70.0 ± 3.3%) and ringtail (62.2 ± 10.2%) were similar. For *A. pungens*, only the seeds dispersed by the gray fox germinated at a very low percentage (1.1 ± 1.9%) similar to the germination percentage in the control seeds (2.2 ± 1.9%). There were therefore no statistical differences found (*F*_2,4_ = 0.57, *p* = .60). Finally, in *Juniperus* sp., we observed that the percentages of germination in seeds dispersed by the gray fox (12.2 ± 8.4%) and ringtail (17.5 ± 10.6%) were higher than that of the control (5.6 ± 1.9%), although these differences were not significant (*F*_4,5_ = 1.79, *p* = .27). In the tropical dry forest, for *F. phillyreoides*, we found significant differences in the average germination percentages of seeds dispersed by each mammal compared to the control (*F*_2,6_ = 14.16, *p* < .0001), with a higher average percentage for the control seeds (73.3 ± 3.3%) than in the seeds dispersed by any of the mammals, but particularly the ringtail, for which the seeds had the lowest germination percentage (27.8 ± 5.1%). On the other hand, for *M. geometrizans*, there were no significant differences between the average germination (±*SD*) of the control and that of the seeds from the animals (*F*_3,8_ = 0.65, *p* = .60). However, the seeds dispersed by the gray fox presented greater germination (58.9 ± 20.4%) than the control seeds (44.4 ± 6.9%) (Table 3).

**TABLE 3:** Average germination percentages ($\bar{x}$ ± *SD*) of seeds of *Arbutus* sp., *Arctostaphylos pungens*, and *Juniperus* sp., with their respective animal dispersers, in the temperate forest, and of seeds of *Forestiera phillyreoides* and *Myrtillocactus geometrizans*, with their respective animal dispersers, in the tropical dry forest (both forests located within the Sierra Fría PNA in Aguascalientes, Mexico)

Forest	Seed species	Disperser species	Seeds (*N*)	Germination (%)
Temperate	*Arbutus* sp.	Ringtail	90	62.2 ± 10.2
		Control (canopy)	90	70.0 ± 3.3
	*A. pungens*	Gray fox	90	1.1 ± 1.9
		Coyote	2	0.0
		Control (canopy)	90	2.2 ± 1.9
	*Juniperus* sp.	Gray fox	90	12.2 ± 8.4
		Coyote	6	0.0
		Ringtail	40	17.5 ± 10.6
		Bobcat	11	0.0
		Control (canopy)	90	5.6 ± 1.9
Tropical	*F. phillyreoides*	Gray fox	90	49.8 ± 17.1
		Ringtail	90	27.8 ± 5.1*
		Control (canopy)	90	73.3 ± 3.3
	*M. geometrizans*	Gray fox	90	58.9 ± 20.4
		Ringtail	90	52.2 ± 3.9
		Coati	90	50.0 ± 13.3
		Control (canopy)	90	44.4 ± 6.9

Note

*N* indicates the maximum number of seeds per treatment.

* Statistically significant differences according to the Dunnett test (*p* < .05).

## 4 DISCUSSION

During the study period, we found scats of gray fox, coyote, ringtail, coati, and bobcat, which are associated with seed dispersal in the temperate and the tropical dry forest, while plants dispersed by mammals are widely distributed in the temperate zone of the PNA‐SF (Díaz‐Núñez et al., 2016). This is the case for *A. pungens*, the seeds of which were dispersed at a higher average abundance in the scats of gray fox, which corroborate previous findings for the same species (e.g., Rubalcava‐Castillo et al., 2020). It is important to mention the role of the bobcat as a diploendozoochoric seed disperser (Hämäläinen et al., 2017), since *J. deppeana* seeds were found in its scats (Rubalcava‐Castillo et al., 2020). In the tropical dry forest, the coati dispersed the highest abundance of seeds, with more than 8,600 seeds in only three scats. Coatis can therefore play a key role in maintaining the dispersal service by spreading large amounts of seeds (Alves‐Costa & Eterovick, 2007). The richness of the plant species found in the scats of the four dispersing carnivores for the tropical dry forest was higher than in the temperate forest. Although only five dispersed plant species were found in the tropical forest, suggesting that, despite the great richness of plant species in our study area (Argumedo‐Espinoza et al., 2018), the mammals selectively feed on only a few plant species (Koike et al., 2008). Neither endozoochory nor diploendozoochory affected the viability or germination of the seed species in the two forest types. These results suggest that the viability of the seeds dispersed by the mammals under study was unaffected, which is crucial for retention (Nogales et al., 2015), the production of holes and cracks in the seed testas (Costea et al., 2016) and for improving the selective germination of thick‐testa seeds in temperate forest and thin‐testa seeds in tropical dry forest.

### 4.1 Seed dispersal

In the temperate forest, the gray fox was the most efficient mammal since 100% of its scats contained seeds of some plant species, demonstrating the ability of this animal to adapt its eating habits to consumption of fruits as an important resource in its diet (Valkenburgh, 1996). However, seed dispersal depending may vary among different species of fox and the plant species. For instance, Bravo et al. (2019) report that 46% of the scats of the Andean fox (*Lycalopex culpaeus*) contained seeds of *Prunus cerasus* and *Malus domestica*. Nevertheless, foxes may be one of the main vectors of dispersal in forests.

While plants dispersed by mammals are widely distributed in the temperate of the PNA‐SF (Díaz‐Núñez et al., 2016), the abundance of dispersed or endozoochoric seeds may vary depending on region. In our case, the seeds of *A. pungens*, the seeds of which were dispersed at a higher average abundance in the scats of gray fox, which corroborate previous findings (e.g., Rubalcava‐Castillo et al., 2020). However, Matías et al. (2010) reported a very low average abundance of *A. uva‐ursi* seeds dispersed by red fox (*Vulpes vulpes*), marten (*Martes foina*), and wild boar (*Sus scrofa*). The seeds of *Arbutus* sp. were only dispersed by the ringtail, so dispersal of this plant species in the temperate forest of our region could be attributed solely to this mammal through selective feeding on the fruits of this plant (Koike et al., 2008). Long‐term future studies are therefore important to our understanding of the variables that influence the dispersal of seeds in the different regions, the animal species involved and their preference for the ingestion of certain species of fruits.

It is important to mention the role of the bobcat as a diploendozoochoric seed disperser (Hämäläinen et al., 2017), since *J. deppeana* seeds were found in its scats, similar to the findings of Rubalcava‐Castillo et al. (2020). Our results therefore reinforce those of other studies that demonstrate the diploendozoochory in some of these carnivores (Kurek & Holeksa, 2015; Sarasola et al., 2016). For this reason, it is important to consider hypercarnivores, such as bobcats, as an important component of the guild of seed dispersers.

In the tropical dry forest, the mammals under study actively participated in seed dispersal by spreading many seeds across the landscape. Such is the case of the coati, which dispersed the highest abundance of seeds, with more than 8,600 seeds of the species *M. geometrizans* and *P. laevigata* found in only three scats. Coatis can therefore play a key role in maintaining the dispersal service by spreading large amounts of seeds (Alves‐Costa & Eterovick, 2007). Likewise, the gray fox and the ringtail dispersed a large number of seeds of a greater variety of plant species and therefore play a role as alternative dispersers in forest landscapes such as that of the tropical dry forest (Alves‐Costa & Eterovick, 2007).

The richness of the plant species found in the scats of the four seed‐dispersing carnivores for tropical dry forest was higher than in temperate forest, as verified in the multivariate GLM analysis, although only five dispersed plant species were found. This is a low amount when compared to other areas of tropical forest where the richness of species dispersed by carnivores is higher (Alves‐Costa & Eterovick, 2007; Zarco‐Mendoza et al., 2018) suggesting that, despite the great richness of plant species in our study area (Argumedo‐Espinoza et al., 2018), the gray fox, ringtail, coati, and badger selectively feed on only a few plant species (Koike et al., 2008).

### 4.2 Viability

Viability is an essential property for seed germination, survival, and establishment. In the temperate forest, most of the *A. pungens* seeds remained viable after being dispersed by the gray fox and ringtail, showing that passage of these seeds through the tract of these mammals did not affect their viability. In contrast, Rubalcava‐Castillo et al. (2020) observed reduced viability of this plant species after being dispersed by the gray fox and ringtail. This contrasting finding suggests that the alterations or damage that the seeds undergo in the digestive tract may differ according to the year and study area, since these are the same plant and mammal species. Likewise, the seeds of *Arbutus* sp. dispersed by the ringtail seem to be unaffected by dispersion through endozoochory, since most of the seeds remained viable. However, there are other factors associated with dispersal as well as viability, such as the abundance, dispersal distance, and germination of dispersed seeds, that must be addressed before this mammal can be considered an effective agent for dispersal of this plant species.

Diploendozoochory produced a low percentage of viability in the seeds dispersed by the bobcat. Conversely, Nogales et al. (2015) verified that the viability of *J. turbinata* seeds by diploendozoochory in the *Galliota* lizard and its predator, the feline *Felis*, remained quite high. The decrease in viability of seeds associated with the bobcat could therefore be due to several factors: (A) possible damage to the embryos as a consequence of the high retention times in the digestive tracts of the carnivores (Varela & Bucher, 2006), (B) possible damage caused from the initial disperser/prey (in this case the rabbit), or (C) the seeds collected from the canopy were defective.

In the tropical dry forest, the seeds of *F. phillyreoides* presented the highest viability in the scats of the gray fox, similar to the findings of Campos and Ojeda (1997) on the viability of *P. flexuosa* seeds from gray fox scats. This suggests that this mammal can disperse different forest species without negatively affecting the viability of the seeds. For *M. geometrizans*, the majority of seeds were able to remain viable in coati scats, similar to that observed by Alves‐Costa and Eterovick (2007), who reported that the seeds can remain viable after passing through the digestive tract of this mammal. This establishes the role and importance of the coati as a mammal capable of dispersing a high quantity of *M. geometrizans* seeds without affecting viability.

### 4.3 Wear of the testa thickness

The result of the dispersal process involving passage through the animal gut could be aided by the seeds coming in contact with digestive tract acids, which cause changes in the internal and external structures of the testas, generally decreasing their thickness (Nogales et al., 2007, 2015; Traveset et al., 2001). We had expected reduced thickness in the seeds dispersed by the animals; however, the opposite was found. The fact that the seed coat is thinner in the undigested seeds of the canopy relative to the seeds in the scats of all the dispersers could indicate that the seed coat swells in some way while passing through the digestive tract. The thickness of the testas was thicker in the seeds of *Juniperus* sp. and *F. phillyreoides* that were found in the scats of all the animal species, especially those of the ringtail. In our case, the increase in thickness might be due to: (a) the length of time that the seeds remained in the tracts, and the absorption of liquids in the intestines thus causing swelling of the seeds, (b) the selection of fruits for the controls, that is, seed thickness may vary according to the selected tree and even the year and time of collection, such that the seeds selected for this study for some reason may have had a thinner testa layer than the average, or (c) the dispersers may select/transport seeds that have thicker testa layers on average. However, it was not possible to demonstrate this in the present study, and subsequent studies should aim to describe this absorption of liquids by seed coatings caused by the passage of the seeds through the tracts of mammals and perform an analysis of the controls through the seasons to establish whether such variation in thickness does in fact occur.

Through scanning electron microscopy, we are also possible observed the removal of the superficial vegetal layers, as well as cracks between the internal and external structures of the testa of seeds of some of the plant species that passed through the animal gut, such as *Juniperus* sp. and *F. phillyreoides*. This is similar to the case of *Cuscuta* seeds that passed through the digestive tracts of aquatic birds (Costea et al., 2016) as well as for *Vaccinium myrtillus* dispersed by the mammal *M. foina* (Schaumann & Heinken, 2002). The removal and fragmentation of the testa probably benefitted the seeds by increasing their permeability to essential elements (water, light, oxygen) for germination. We can, thus, conclude that most of these seeds that passed through their digestive tracts of the carnivores remain viable and undergo a process of production of holes and cracks in the testas that can facilitate the entry of water and oxygen, which could benefit their subsequent germination.

### 4.4 Germination

The carnivores in this study had varying impacts on the germination of the seeds they dispersed depending on the plant species. Although the action of the ringtail did not lead to a higher germination rate than that of the control in *Arbutus* sp. seeds, the percentages were very similar. This suggests that the passage of seeds of this plant species through the tract of ringtail is adapted to the process of endozoochory since the germination of the seeds was not affected. The seeds of this genus must be freed from the pulp of the fruit to successfully germinate (Narbona et al., 2003), which is then enabled by endozoochory. The seeds of *A. pungens* presented very low germination in the gray fox scats, although the values are higher than the zero‐germination observed by Rubalcava‐Castillo et al. (2020) for seeds planted under the same temperature conditions and incubation in a germination chamber. Likewise, Rubalcava‐Castillo et al. (2020) obtained lower germination rates in seeds of *J. deppeana* dispersed by gray fox, coyote, and bobcat, compared to the rates we report in the present study for gray fox and ringtail. This might be an indication of how the alterations to the testas of the *Juniperus* sp. seeds could have caused an increase in germination rates.

The seeds dispersed through diploendozoochory by the bobcat failed to germinate, possibly because some vertebrates with strong enzymatic digestion, such as the felines, actually damaged the seeds (Nogales et al., 2015). However, Rubalcava‐Castillo et al. (2020) reported the germination of *Juniperus* sp. seeds found in bobcat scats. Due to these variable rates of germination and high variation in seed viability of some *Juniperus* species (Rumeu et al., 2011), it is difficult to evaluate the effect of felines on their germination (Nogales et al., 2015) because the potential impacts of the primary disperser must also be considered, as well as the fact that the high latency recorded must be integrated with the low germination described for many *Juniperus* species (Adams, 2008; Rumeu et al. 2009).

In the tropical dry forest, for the seeds of *F. phillyreoides*, the highest percentages of germination were presented by the control seeds. Despite this, the seeds of *F. phillyreoides* dispersed by the gray fox presented a higher percentage compared with the other animals, and seeds dispersed by the gray fox can therefore remain viable without improving germination relative to the controls (Campos & Ojeda, 1997). Passage through the digestive tract can thus have a positive, neutral, or negative effect on germination (Cypher & Cypher, 1999), and the adaptive importance of these mammals to trees such as *F. phillyreoides* is consequently related to dispersal (Peguero & Espelta, 2014). In addition, these animals can also be scarifiers of *F. phillyreoides* seeds, which benefits from this pregerminative treatment through removal of the endocarp (Martínez‐Calderón et al., 2020). The seeds of *M. geometrizans* found in the scats of all of the mammals presented germination rates greater than that of the control, particularly in seeds dispersed by the gray fox, which was the animal species found to have the greatest influence on seed germination (Traba et al., 2006).

## 5 CONCLUSIONS

The study showed that, in both the temperate and tropical dry forests, carnivores consuming fruits provide important seed dispersal services, by defecating viable seeds that are able germinate and thus can extend and reinforce the forest structure. However, the abundance and richness of dispersed seeds varies according the type of forest in which the dispersers are found. Seeds with thick testa in temperate forest and those with thin testa in tropical dry forest seem to be adapted to scarification by endozoochory, where mammals generate structural changes and openings in the testas, improving germination without affecting viability. In this study, the abundant and efficient participation of the gray fox and ringtail as dispersers of *Arctostaphylos pungens* in the temperate forest, and *Myrtillocactus geometrizans* in the tropical dry forest was highlighted. However, bobcat diploendozoochory acted to preserve seed viability, without improving germination. These results suggest that carnivores can perform an important ecological function by dispersing a great abundance of seeds, scarifying these seeds causing the formation of holes and cracks in the testas without affecting viability and promoting the selective germination of seeds.

## CONFLICT OF INTEREST

No authors disclose any conflict of interest.

## AUTHOR CONTRIBUTIONS

**Fabián Alejandro Rubalcava‐Castillo:** Conceptualization (lead); data curation (lead); formal analysis (lead); investigation (lead); methodology (lead); project administration (lead); software (lead); supervision (lead); validation (lead); visualization (lead); writing‐original draft (lead); writing‐review & editing (lead). **Joaquín Sosa‐Ramírez:** Conceptualization (equal); funding acquisition (lead); investigation (equal); methodology (equal); project administration (equal); resources (lead); supervision (lead); validation (lead); writing‐original draft (equal); writing‐review & editing (equal). **José de Jesús Luna‐Ruíz:** Conceptualization (equal); data curation (equal); formal analysis (equal); investigation (equal); methodology (equal); project administration (equal); supervision (equal); writing‐original draft (equal); writing‐review & editing (equal). **Arturo Gerardo Valdivia‐Flores:** Conceptualization (equal); data curation (equal); formal analysis (equal); investigation (equal); methodology (equal); project administration (equal); software (equal); supervision (equal); validation (equal); visualization (equal); writing‐original draft (equal); writing‐review & editing (equal). **Luis Ignacio Íñiguez‐Dávalos:** Conceptualization (equal); investigation (equal); methodology (equal); project administration (equal); validation (equal); writing‐original draft (equal); writing‐review & editing (equal).

## Data Availability

The sampling locations for each scat are available on Dryad (https://doi.org/10.5061/dryad.c866t1g5m).
